# Clinical and Treatment-Related Factors Associated with Neutrophil Recovery After Bloodstream Infection in Acute Leukemia: A Competing-Risk Analysis

**DOI:** 10.3390/cancers18193234

**Published:** 2026-10-08

**Authors:** Krzysztof Gawronski, Nadia Hussein, Piotr Rzepecki, Aneta Guzek

**Affiliations:** 1 Department of Hematology and Internal Medicine, Military Institute of Medicine–National Research Institute, 128 Szaserów Street, 04-141 Warsaw, Poland; 2 Department of Laboratory Diagnostics, Military Institute of Medicine–National Research Institute, 128 Szaserów Street, 04-141 Warsaw, Poland

**Keywords:** acute leukemia, bloodstream infection, neutrophil recovery, competing risks, G-CSF, antimicrobial resistance

## Abstract

Background/Objectives: Neutrophil recovery after bloodstream infection (BSI) in acute leukemia is clinically important, but evaluation is complicated by early death and recurrent infectious episodes. We assessed clinical, treatment-related, and microbiological factors associated with recovery while accounting for death as a competing event. Methods: This retrospective single-center cohort included 205 microbiologically documented BSI episodes in 101 adults with acute leukemia treated between 2020 and 2025. Neutrophil recovery was defined as an absolute neutrophil count >500/µL after BSI onset. The endpoint was evaluable in 186 episodes; 116 were followed by recovery and 70 by death before recovery. Recurrent episodes were retained and within-patient dependence was addressed by patient-level clustering in marginal Fine–Gray regression. Results: In the fully adjusted model (177 episodes, 93 patients), G-CSF use was associated with a higher cumulative incidence of recovery (sHR 3.17, 95% CI 1.40–7.18; *p* = 0.006), as was consolidation versus induction (sHR 2.50, 95% CI 1.44–4.33; *p* = 0.001). Clinically relevant antimicrobial resistance was not independently associated with recovery (sHR 0.80, 95% CI 0.38–1.68; *p* = 0.558). A literal-first-BSI sensitivity analysis supported the overall clinical pattern. Conclusions: Neutrophil recovery after BSI was associated primarily with treatment and clinical context, while the crude adverse association with antimicrobial resistance was substantially attenuated after adjustment. These observational associations, particularly those involving G-CSF, should not be interpreted causally.

## Simple Summary

Patients with acute leukemia frequently develop bloodstream infections during profound neutropenia. Neutrophil recovery is clinically important, but some patients die before recovery and others experience repeated infections. We analyzed 205 bloodstream infection episodes in 101 adults using a competing-risk approach that accounted for death before recovery and recurrent infections in the same patient. Recovery was more frequent during consolidation treatment and in episodes in which granulocyte colony-stimulating factor (G-CSF) was used. Antimicrobial resistance was associated with poorer recovery in unadjusted analysis, but this association largely disappeared after treatment context, infection severity, inflammation, and supportive care were considered together. These findings suggest that recovery after bloodstream infection reflects the broader clinical and treatment setting rather than antimicrobial resistance alone. Because this was a retrospective observational study, the association with G-CSF should not be interpreted as proof of a treatment effect.

## 1. Introduction

Bloodstream infections (BSIs) are among the most serious complications of intensive treatment for acute leukemia. Profound and prolonged neutropenia, disruption of mucosal barriers, repeated hospitalization, and exposure to broad-spectrum antimicrobial agents contribute to both the occurrence and severity of these infections [1]. Antimicrobial prophylaxis is recommended for selected patients expected to experience profound and protracted treatment-related neutropenia [2]. Although mortality remains the principal outcome evaluated in studies of BSI in patients with hematologic malignancies, restoration of neutrophil production represents another clinically important component of recovery. Failure to regain effective neutrophil production may prolong susceptibility to infection, increase antimicrobial exposure and hospitalization, and interfere with subsequent antileukemic treatment [1].

Neutrophil recovery after intensive chemotherapy is shaped by multiple interacting factors. The underlying leukemia and treatment context influence the depth and duration of marrow suppression, while systemic inflammation and severe infection may further alter the clinical course. Granulocyte colony-stimulating factor (G-CSF) can shorten the duration of neutropenia after intensive AML therapy [3], but its use in routine clinical practice is influenced by treatment context and patient condition. Consequently, the probability of neutrophil recovery following BSI cannot be attributed to a single infectious or therapeutic factor.

We previously showed that infectious complications and neutropenic fever were associated with prolonged hematopoietic recovery after induction or consolidation chemotherapy in patients with acute leukemia [4]. That study compared recovery times among patients in whom hematopoietic recovery could be assessed and excluded patients who died before recovery; it therefore did not address recovery in the presence of competing mortality or recurrent BSI episodes.

An important unanswered question is therefore which characteristics of the BSI episode and its clinical context are associated with subsequent neutrophil recovery when death before recovery is explicitly considered. Antimicrobial resistance, septic shock, systemic inflammation, polymicrobial infection, treatment context, and supportive use of G-CSF may all influence the observed probability of recovery. Their relative contributions, however, are difficult to distinguish using analyses restricted to survivors or conventional comparisons of recovery times. This problem is further complicated by recurrent BSI, because individual patients may experience multiple infectious episodes during different phases of leukemia treatment.

The present study therefore evaluated neutrophil recovery following microbiologically documented BSI in patients with acute leukemia using a competing-risk approach, with death before recovery treated as a competing event and recurrent BSI accounted for at the patient level. We examined whether treatment context, G-CSF use, infection severity, systemic inflammation, and clinically relevant antimicrobial resistance were associated with the cumulative incidence of neutrophil recovery. We further assessed whether the crude association between antimicrobial resistance and recovery persisted after adjustment for treatment- and episode-related clinical factors.

## 2. Materials and Methods

### 2.1. Study Design and Setting

We performed a retrospective single-center cohort analysis of microbiologically confirmed bloodstream infection (BSI) episodes recorded from 2020 through 2025 in adults with acute leukemia treated at the Clinic of Internal Medicine and Hematology, Military Institute of Medicine–National Research Institute, Warsaw, Poland. Each BSI episode served as the analytical unit. Patients with more than one BSI during follow-up could therefore contribute multiple observations; dependence between episodes from the same patient was addressed by patient-level clustering in the primary regression model.

The present analysis uses an institutional population and study period that partially overlap with two previous reports from our center, but the research questions, analytical populations, and statistical approaches are distinct [4,5]. The first report evaluated recovery time after induction or consolidation chemotherapy across groups defined by documented infection, neutropenic fever, or absence of fever/infection; patients who died before recovery were excluded and no multivariable competing-risk model was performed [4]. The second report was a pathogen-specific comparison of NDM-producing Klebsiella pneumoniae BSI versus other pathogens, with mortality as the primary endpoint and hematopoietic recovery as a secondary outcome [5]. In contrast, the present study evaluates determinants of neutrophil recovery across microbiologically documented BSI episodes, retains recurrent infections, treats death before recovery as a competing event, and jointly models treatment context, G-CSF exposure, infection severity, systemic inflammation, and clinically relevant antimicrobial resistance with patient-level clustering.

Clinical and microbiological information was obtained retrospectively from routinely collected records. The study protocol was approved by the Bioethics Committee of the Military Institute of Medicine–National Research Institute (approval No. 55/26). The requirement for individual informed consent was waived because the study involved retrospective analysis of anonymized data.

### 2.2. Study Population and BSI Episodes

The cohort comprised microbiologically confirmed BSI episodes occurring in patients with acute myeloid leukemia (AML), including acute megakaryoblastic leukemia, or acute lymphoblastic leukemia (ALL), including B-cell ALL and T-cell ALL. The descriptive dataset contained 205 episodes from 101 patients. Recurrent infections were analyzed as separate episodes because treatment context, inflammatory and clinical status, microbiological findings, and the subsequent course of neutrophil recovery could vary between infections experienced by the same patient. No formal sample-size calculation was performed; the study size was determined by all BSI episodes meeting the eligibility criteria that were identified in the study records during the prespecified 2020–2025 study period. Acute megakaryoblastic leukemia was classified within AML rather than as a separate diagnostic category.

### 2.3. Microbiological Procedures

Blood culture bottles were incubated and monitored with the BacT/Alert Virtuo system (bioMérieux, Marcy-l’Étoile, France). Isolates were identified by MALDI-TOF mass spectrometry on the VITEK MS platform (bioMérieux, Marcy-l’Étoile, France), and routine antimicrobial susceptibility testing was performed with VITEK 2 (bioMérieux, Marcy-l’Étoile, France) using the EUCAST interpretive criteria applicable at the time of testing. Extended-spectrum beta-lactamase production was phenotypically confirmed by double-disk synergy testing when required. Carbapenemase detection, including NDM and OXA-48-like enzymes, was performed with RESIST-5 assays (Coris BioConcept, Gembloux, Belgium). Susceptibility to colistin was determined by broth microdilution. For selected positive blood cultures, the BioFire BCID2 panel (BioFire Diagnostics, Salt Lake City, UT, USA) was additionally used for rapid organism identification and detection of relevant resistance markers.

For this study, antimicrobial resistance was grouped according to a prespecified clinically oriented definition. The resistant category included ESBL-producing Enterobacterales; carbapenem-resistant or carbapenemase-producing Enterobacterales, including NDM-, OXA-48-, KPC-, and other metallo-beta-lactamase-associated phenotypes where applicable; vancomycin-resistant enterococci (VRE); and methicillin-resistant Staphylococcus aureus (MRSA). Methicillin resistance in coagulase-negative staphylococci, isolated high-level aminoglycoside resistance, and MLSB resistance in the absence of one of the predefined phenotypes were not counted as clinically relevant resistance for the present analysis.

### 2.4. Clinical Variables and Treatment Context

Episode-level variables included leukemia diagnosis, treatment context at the time of BSI, septic shock, multiple organ dysfunction syndrome (MODS), granulocyte colony-stimulating factor (G-CSF) use, and C-reactive protein (CRP) measured in temporal relation to the BSI episode. Treatment context was originally recorded as induction, consolidation, reinduction, salvage therapy, conditioning regimen, or prolonged therapy. Induction and consolidation represented sequential phases of antileukemic treatment, whereas conditioning represented high-dose preparative therapy administered before hematopoietic stem cell transplantation (HSCT). For multivariable modeling, these categories were grouped into four analytical treatment contexts: induction; consolidation; reinduction/salvage/prolonged therapy; and conditioning for HSCT. Induction served as the reference category. These categories characterized the clinical and therapeutic setting in which BSI occurred and were not intended to compare the efficacy of alternative treatment strategies.

G-CSF use was coded as a binary episode-level exposure. The analytical dataset contained only a yes/no indicator of G-CSF use; dose, schedule, and exact timing relative to BSI onset and ANC recovery were not available for modeling. G-CSF was therefore treated as an observational covariate rather than as a time-dependent treatment exposure. In the primary model, CRP was entered continuously and expressed per 10 mg/dL increase. Septic shock was chosen as the principal marker of infection severity. Because MODS substantially overlapped clinically with septic shock, it was evaluated in a sensitivity analysis rather than entered simultaneously in the primary model.

### 2.5. Neutrophil Recovery Endpoint

The primary outcome was neutrophil recovery after BSI, operationalized as an absolute neutrophil count (ANC) exceeding 500/µL. Follow-up for each episode began on the BSI start date, defined by collection of the positive blood culture, and continued until documented ANC recovery. A death occurring before ANC recovery was considered a competing event rather than a censoring event.

Outcome status could be established for 186 of the 205 BSI episodes. Neutrophil recovery occurred after 116 episodes, whereas 70 episodes were followed by death before recovery. For 19 episodes, available source records did not permit reliable reconstruction of the recovery endpoint. Because no defensible censoring time could be assigned, these observations were omitted from the time-to-event analyses rather than censored or imputed.

### 2.6. Statistical Analysis

Continuous data were described by medians and interquartile ranges (IQRs), whereas categorical data were summarized as counts and percentages. Cumulative incidence functions (CIFs), with death before ANC recovery treated as the competing event, were used for descriptive visualization of recovery according to treatment context, G-CSF use, and septic shock.

Associations with neutrophil recovery were estimated with Fine–Gray regression for the subdistribution hazard [6]. The primary analysis retained recurrent BSI episodes; therefore, correlation among observations contributed by the same patient was accommodated with patient-level clustering and a robust sandwich variance estimator based on the clustered competing-risk framework described by Zhou et al. [7]. Results are presented as subdistribution hazard ratios (sHRs) with 95% confidence intervals (CIs). In this setting, an sHR above 1 denotes a higher cumulative incidence of ANC recovery over time in the presence of competing mortality, whereas an sHR below 1 denotes a lower cumulative incidence. Subdistribution hazard ratios were interpreted in relation to the cumulative incidence of recovery rather than as cause-specific hazards [8].

Multivariable adjustment was organized sequentially according to the prespecified analysis plan rather than data-driven variable selection. Model 1 contained treatment context and G-CSF use. Model 2 added septic shock and CRP, expressed per 10 mg/dL. Model 3 further incorporated clinically relevant antimicrobial resistance and constituted the primary fully adjusted model. Univariable significance was not used as a criterion for entering or removing variables from these models.

Regression models were fitted to complete cases for the covariates included at each stage. Model 1 comprised 180 episodes contributed by 94 patients. Models 2 and 3 comprised 177 episodes from 93 patients, including 112 recovery events and 65 deaths before recovery. Missing covariate values were not imputed.

### 2.7. Sensitivity and Model-Diagnostic Analyses

Robustness was examined using prespecified sensitivity analyses. First, the fully adjusted model was repeated using only the literal chronological first BSI experienced by each patient. Additional analyses substituted MODS for septic shock, polymicrobial BSI for the resistance variable, and log2-transformed CRP for the linear CRP term. For the first-BSI analysis, the earliest episode was selected for each of the 101 patients; 89 first episodes had an evaluable recovery endpoint and 84 had complete data for all covariates in the fully adjusted model. As each patient contributed only one observation to this analysis, a conventional independent Fine–Gray model was used without cluster-robust variance estimation.

Covariate proportionality was also examined diagnostically. The diagnostic assessment suggested possible time variation in the CRP association; consequently, a time-varying CRP model was explored as a secondary analysis. This diagnostic analysis was not part of model selection and did not modify the prespecified primary Model 3.

All statistical tests were two-sided, and *p* < 0.05 was considered statistically significant. Descriptive analyses and data processing were performed using TIBCO Statistica, version 13 (TIBCO Software Inc., Palo Alto, CA, USA). Competing-risk analyses followed the Fine–Gray framework and the clustered extension described above [6,7,8].

### 2.8. Missing Data

Missing data were handled using available-case summaries and complete-case regression. The 19 episodes for which the neutrophil-recovery endpoint could not be reconstructed were excluded from time-to-event analyses. Among the remaining 186 episodes, treatment context was missing for four episodes, G-CSF status for three, and CRP for four, with some missing values occurring in the same observations. Accordingly, 177 episodes entered the fully adjusted primary model. Neither outcome nor covariate values were imputed.

### 2.9. Use of Generative AI and AI-Assisted Technologies

Generative artificial intelligence (AI) and AI-assisted technologies were not used at any stage in the preparation, revision, analysis, writing, editing, or formatting of this manuscript.

## 3. Results

### 3.1. Study Cohort and Hematopoietic Recovery

The study cohort comprised 205 microbiologically documented BSI episodes in 101 patients with acute leukemia. The neutrophil-recovery endpoint was evaluable for 186 episodes (90.7%). ANC recovery to >500/µL occurred in 116 episodes, whereas 70 episodes ended in death before recovery and were treated as competing events. In 19 episodes, the recovery endpoint could not be reliably established from the source data; these episodes were retained for overall descriptive analyses but excluded from time-to-event analyses. Among the 186 endpoint-evaluable episodes, the observed time from BSI onset to ANC recovery or competing death was a median of 11.0 days (IQR, 5.3–19.8 days).

Clinical and microbiological characteristics differed substantially across the two observed outcome states (Table 1). Septic shock was present in 23/116 (19.8%) episodes followed by ANC recovery and 31/70 (44.3%) episodes ending in death before recovery. MODS occurred in 10/116 (8.6%) and 20/70 (28.6%) episodes, respectively. Clinically relevant resistant phenotypes were identified in 22/116 (19.0%) recovery episodes and 29/70 (41.4%) competing-death episodes, while polymicrobial BSI occurred in 14/116 (12.1%) and 18/70 (25.7%), respectively. Median CRP was 5.7 mg/dL (IQR 3.1–11.2) in episodes followed by recovery and 13.4 mg/dL (IQR 8.8–24.1) in episodes ending in death before recovery.

To assess potential attrition bias related to the 19 episodes with an unreconstructable recovery endpoint, we compared key available baseline and episode characteristics with those of the 186 endpoint-evaluable episodes. Septic shock occurred in 3/19 (15.8%) versus 54/186 (29.0%) episodes, MODS in 0/19 (0%) versus 30/186 (16.1%), clinically relevant antimicrobial resistance in 3/19 (15.8%) versus 51/186 (27.4%), and polymicrobial BSI in 1/19 (5.3%) versus 32/186 (17.2%), respectively. Median CRP was 6.8 mg/dL among excluded episodes and 8.5 mg/dL among endpoint-evaluable episodes. Although these descriptive comparisons do not suggest preferential exclusion of episodes with greater acute infectious severity, the small excluded group and differences in treatment-context distribution preclude assuming that endpoint unavailability was random. Detailed comparisons are provided in Supplementary Table S2.

Because G-CSF exposure was clinically selected rather than assigned, we also compared its distribution across treatment context and markers of acute infection severity. Among 199 episodes with a clear G-CSF status, 55 were G-CSF-exposed and 144 were unexposed. Conditioning for HSCT accounted for 31/55 (56.4%) G-CSF-exposed episodes but only 2/144 (1.4%) unexposed episodes. Septic shock occurred in 16/55 (29.1%) versus 40/144 (27.8%), MODS in 10/55 (18.2%) versus 19/144 (13.2%), and median CRP was 8.0 versus 8.6 mg/dL, respectively. Thus, recorded G-CSF exposure was strongly dependent on treatment context, particularly the transplant-related setting, whereas measured acute infectious severity was broadly similar between exposure groups. The full descriptive comparison is provided in Supplementary Table S1.

### 3.2. Cumulative Incidence of ANC Recovery

The cumulative incidence of ANC recovery varied according to treatment context, G-CSF use, and clinical severity (Figure 1). By day 30, recovery had occurred in 88.6% of consolidation episodes, 54.5% of induction episodes, 70.6% of episodes during conditioning, and 31.6% of episodes during reinduction, salvage, or prolonged therapy. The corresponding 30-day cumulative incidence was 74.5% among episodes with G-CSF use and 55.3% among episodes without G-CSF. For septic shock, 30-day recovery was 42.6%, compared with 67.4% in episodes without septic shock. These curves are descriptive; formal inference accounting for repeated BSI episodes was based on clustered competing-risk regression.

### 3.3. Competing-Risk Regression

To determine which treatment- and infection-related factors were independently associated with neutrophil recovery in the presence of competing mortality, sequential patient-clustered Fine–Gray regression models were fitted. Overall, treatment context and recorded G-CSF exposure showed the most consistent associations with recovery, whereas the crude adverse associations of infection severity, systemic inflammation, and antimicrobial resistance were substantially attenuated after multivariable adjustment.

In Model 1, which included treatment context and G-CSF, consolidation versus induction was associated with a higher recovery incidence (sHR 3.21, 95% CI 1.84–5.62; *p* < 0.001), and G-CSF use was also associated with higher recovery (sHR 2.80, 95% CI 1.24–6.35; *p* = 0.014). After addition of septic shock and CRP in Model 2, both consolidation (sHR 2.63, 95% CI 1.53–4.53; *p* < 0.001) and G-CSF use (sHR 3.25, 95% CI 1.42–7.42; *p* = 0.005) remained associated with higher recovery. Higher CRP showed a modest inverse association in Model 2 (sHR 0.72 per 10 mg/dL, 95% CI 0.53–0.98; *p* = 0.036).

The fully adjusted Model 3 included 177 BSI episodes from 93 patients, with 112 ANC recovery events and 65 competing deaths (Table 2). G-CSF use remained associated with a higher subdistribution hazard of recovery (sHR 3.17, 95% CI 1.40–7.18; *p* = 0.006), and consolidation versus induction was also associated with higher recovery (sHR 2.50, 95% CI 1.44–4.33; *p* = 0.001). Reinduction, salvage, or prolonged therapy (sHR 0.54, 95% CI 0.25–1.21; *p* = 0.135), conditioning (sHR 0.62, 95% CI 0.26–1.47; *p* = 0.280), septic shock (sHR 0.73, 95% CI 0.35–1.54; *p* = 0.414), and CRP (sHR 0.73 per 10 mg/dL, 95% CI 0.53–1.00; *p* = 0.053) were not independently associated with recovery at the conventional 0.05 threshold in this model. In an additional model contrast performed during revision, consolidation was associated with a higher subdistribution hazard of neutrophil recovery than conditioning for HSCT (sHR 4.03, 95% CI 1.66–9.76; *p* = 0.002). This contrast represents a comparison between distinct clinical treatment contexts and should not be interpreted as comparative treatment efficacy.

The univariable association between clinically relevant antimicrobial resistance and reduced recovery was markedly attenuated after adjustment. In Model 3, resistance was not independently associated with ANC recovery (sHR 0.80, 95% CI 0.38–1.68; *p* = 0.558).

### 3.4. Sensitivity Analyses

Sensitivity analyses were performed to assess the robustness of the primary findings to restriction to one BSI per patient, alternative characterization of infection severity and microbiological complexity, and alternative modeling of CRP (Table 3). Overall, the principal pattern was preserved across these analyses, particularly the positive associations of consolidation treatment context and recorded G-CSF exposure with recovery.

Replacing septic shock with MODS did not materially alter the main pattern: G-CSF use remained associated with higher recovery (sHR 3.12, 95% CI 1.42–6.85; *p* = 0.005), whereas MODS showed a direction toward lower recovery without statistical significance (sHR 0.48, 95% CI 0.14–1.64; *p* = 0.240). In this model, consolidation versus induction remained associated with higher recovery (sHR 2.40, 95% CI 1.40–4.10; *p* = 0.001), and clinically relevant resistance remained non-significant (sHR 0.73, 95% CI 0.37–1.46; *p* = 0.376). When polymicrobial BSI replaced resistance, polymicrobial infection was associated with a lower subdistribution hazard of recovery (sHR 0.50, 95% CI 0.27–0.93; *p* = 0.028), while G-CSF remained positively associated with recovery (sHR 3.40, 95% CI 1.56–7.42; *p* = 0.002). Modeling CRP on the log2 scale also left the principal findings unchanged.

### 3.5. Exploratory Assessment of Time Dependence

Exploratory diagnostics suggested possible time dependence of the CRP association, whereas no clear time-dependence signal was observed for treatment context, septic shock, or antimicrobial resistance. In the time-varying CRP sensitivity analysis, the estimated association became progressively more inverse at later time points (sHR per 10 mg/dL approximately 0.79 at day 7, 0.64 at day 14, and 0.51 at day 30). Because this analysis was prompted by model diagnostics rather than prespecified, it was treated as exploratory and did not alter the prespecified linear CRP term in Model 3.

## 4. Discussion

### 4.1. Principal Findings

In this competing-risk analysis of recurrent bloodstream infections in patients with acute leukemia, neutrophil recovery was associated with treatment and clinical context, whereas the crude adverse association with antimicrobial resistance was substantially attenuated after adjustment. G-CSF use and consolidation treatment context showed positive associations with recovery in the fully adjusted clustered model, and both findings were supported by the literal-first-BSI sensitivity analysis. In univariable analyses, septic shock, higher CRP, polymicrobial BSI, and clinically relevant antimicrobial resistance were associated with lower recovery; after multivariable adjustment, these episode-level adverse associations were attenuated.

These findings suggest that neutrophil recovery following BSI should not be viewed solely as a consequence of the microbiological characteristics of the infection. Rather, it appears to reflect the interaction between underlying disease and treatment context, supportive care, and the clinical severity of the infectious episode.

### 4.2. Treatment Context and G-CSF

Treatment context showed a pronounced association with neutrophil recovery. Consolidation was associated with a higher cumulative incidence of recovery than induction. Induction and consolidation are sequential phases of antileukemic therapy, but they occur in clinically and biologically different settings; patients receiving consolidation have generally achieved sufficient disease control to proceed beyond induction. Reinduction and salvage strategies, in contrast, are used in patients with inadequate response, refractory disease, or relapse [9], and contemporary AML cohorts show that infectious burden differs across treatment phases and chemotherapy regimens [10,11,12]. Treatment context may therefore capture differences in leukemia status, marrow reserve, previous treatment exposure, and expected hematopoietic recovery that were not fully measurable in this retrospective dataset. Conditioning should be distinguished from these conventional phases of leukemia-directed chemotherapy because it represents high-dose preparative therapy before HSCT. Accordingly, the consolidation-versus-conditioning contrast should be interpreted as a comparison between distinct clinical treatment contexts rather than as comparative treatment efficacy.

Recorded G-CSF exposure was also associated with a higher cumulative incidence of neutrophil recovery in the adjusted model and in the literal-first-BSI sensitivity analysis. This finding should be interpreted as an observational association rather than evidence of treatment efficacy [13,14]. Importantly, the 2022 European LeukemiaNet recommendations do not advocate routine use of myeloid growth factors in AML; they reserve their use for selected individual patients, such as those with severe infection, or for particular treatment settings, including shortening hematologic recovery during consolidation [9]. This selective clinical use is directly relevant to interpretation of our findings. G-CSF administration in the present cohort was clinically determined rather than randomly assigned, and only a binary episode-level exposure indicator was available; exact timing relative to BSI onset and ANC recovery, dose, duration, and indication were not available for time-dependent modeling. Confounding by indication, reverse causation, and time-related exposure misclassification, including potential immortal-time bias, therefore cannot be excluded. Moreover, G-CSF exposure was strongly concentrated in the conditioning/HSCT context, indicating that it cannot be considered an isolated treatment exposure in this dataset. Residual confounding by transplantation-related factors, graft characteristics, timing of stem-cell infusion, and post-transplant supportive care may therefore persist despite multivariable adjustment.

### 4.3. Clinical Severity and Systemic Inflammation

Septic shock and MODS were markedly more frequent among episodes ending in death before neutrophil recovery than among episodes followed by recovery. In the primary clustered model, septic shock retained a direction toward reduced recovery, although the confidence interval included unity. Importantly, in the literal-first-BSI sensitivity analysis, septic shock was associated with a substantially lower subdistribution hazard of recovery.

Previous studies of BSI in acute leukemia have identified septic shock as an important predictor of early mortality [15,16]. Contemporary cohorts of hematological malignancies and AML also confirm the close relationship between neutropenia, severe BSI, antimicrobial resistance, and adverse clinical outcomes [17,18]. The present analysis approaches the same clinical phenomenon from a different perspective. When death before neutrophil recovery is treated as a competing event, severe infection can reduce the observed probability of neutrophil recovery because early death precludes observation of the recovery endpoint.

CRP was substantially higher in episodes ending in death before recovery than in those followed by recovery. In Model 2, higher CRP showed a modest inverse association with recovery; after addition of antimicrobial resistance in the fully adjusted model, the estimate was slightly attenuated and narrowly missed the conventional threshold for statistical significance (sHR 0.73 per 10 mg/dL, 95% CI 0.53–1.00; *p* = 0.053). Exploratory diagnostics suggested that the association of CRP with recovery may vary over time, with a more inverse point estimate at later follow-up. Longitudinal studies in AML have also demonstrated that CRP dynamics track infectious episodes and broader inflammatory responses during chemotherapy-induced neutropenia [19,20]. This finding should be interpreted cautiously because the time-varying analysis was prompted by model diagnostics rather than prespecified. The exploratory assessment of time dependence was interpreted in accordance with methodological considerations for time-varying terms in Fine–Gray models [21].

### 4.4. Antimicrobial Resistance and Polymicrobial Infection

Clinically relevant antimicrobial resistance was associated with substantially impaired neutrophil recovery in univariable analysis, but this association almost completely disappeared after adjustment. This attenuation is an important finding. Resistant BSI may identify episodes occurring in patients with greater previous healthcare and antimicrobial exposure, more advanced treatment courses, and a higher burden of clinical complications. Consequently, resistance may partly function as a marker of an adverse clinical context rather than as an independent determinant of hematopoietic recovery.

This interpretation does not imply that antimicrobial resistance is clinically unimportant. Resistant and particularly carbapenem-resistant Gram-negative BSI have been associated with increased mortality in patients with acute leukemia, and their adverse consequences may be amplified by inappropriate initial antimicrobial therapy [15,16,22,23,24,25]. Prior antimicrobial exposure has also been linked to subsequent antimicrobial-resistant Gram-negative BSI in AML [26]. Rather, our results suggest that the crude relationship between resistance and neutrophil recovery is substantially explained by the broader treatment and clinical context.

A potentially important secondary finding was the association between polymicrobial BSI and reduced recovery when polymicrobial infection replaced resistance in the fully adjusted sensitivity model (sHR 0.50, 95% CI 0.27–0.93; *p* = 0.028). Polymicrobial BSI has been described as a clinically relevant phenotype in hematological populations [27], with recent data confirming its association with substantial morbidity and mortality in patients with hematologic malignancies [28]. Because our result arose from a sensitivity model and the number of polymicrobial episodes was limited, it should be considered hypothesis-generating rather than definitive.

### 4.5. Importance of Competing Death and Recurrent BSI

An important methodological feature of the present study is the explicit treatment of death before neutrophil recovery as a competing event. Conventional analyses restricted to patients who survive until hematopoietic recovery may preferentially exclude the most severe infectious episodes and thereby produce an overly favorable picture of neutrophil recovery. This issue is particularly relevant in acute leukemia, where severe BSI and profound marrow suppression frequently coexist and early mortality may preclude observation of the hematopoietic endpoint.

The present study also retained recurrent BSI episodes rather than restricting the primary analysis to a single infection per patient. This reflects clinical practice, in which patients with acute leukemia frequently experience repeated infections during different phases of treatment. Recurrent BSI has also been documented in other profoundly immunocompromised hematologic populations, underscoring the clinical relevance of repeated infectious events [29]. Patient-level clustering was incorporated into the primary competing-risk regression to account for within-patient dependence. Because recurrent episodes can also generate complex biological dependence, including overlapping recovery windows, the literal-first-BSI sensitivity analysis provided an important robustness check in which each patient contributed only one observation. The preservation of the principal pattern in that analysis argues against the main findings being driven solely by recurrent or overlapping BSI. More generally, simulation-based comparisons emphasize that competing events and clustering should both be addressed when dependence is present [30].

### 4.6. Strengths and Limitations

The principal strengths of this study include detailed episode-level clinical and microbiological characterization, inclusion of recurrent BSI, explicit consideration of death before recovery as a competing event, and a prespecified sequential modeling strategy separating treatment context, infection severity, systemic inflammation, and antimicrobial resistance. The analysis also distinguishes between crude microbiological associations and associations that persist after accounting for the broader clinical context.

Several limitations should be acknowledged. First, this was a retrospective single-center study with a limited sample size, which restricts causal inference and external generalizability. The cohort was predominantly composed of patients with AML, while ALL and other diagnostic subgroups were small; therefore, applicability beyond AML-dominant populations should be considered cautiously. Second, G-CSF administration was determined clinically rather than randomly, and the analytical dataset captured G-CSF only as a binary episode-level variable without dose, schedule, duration, indication, or exact timing relative to BSI onset and neutrophil recovery. Confounding by indication, reverse causation, time-related exposure misclassification, and potential immortal-time bias therefore cannot be excluded. Third, the number of events limited the precision of some multivariable estimates, particularly for the smaller treatment-context subgroups such as reinduction/salvage/prolonged therapy and conditioning, as reflected by wide confidence intervals. Fourth, the hematopoietic-recovery endpoint could not be reliably reconstructed for 19 BSI episodes; these episodes were excluded rather than imputed because a valid censoring time was unavailable. Fifth, residual confounding by leukemia status, treatment intensity, marrow reserve, transplantation-related factors, or other unmeasured clinical variables remains possible. Finally, some patients contributed several BSI episodes, occasionally with overlapping recovery periods. Although within-patient clustering was incorporated into the primary analysis and a literal-first-BSI sensitivity analysis was performed, the complex biological dependence between successive infectious episodes cannot be fully captured by statistical adjustment.

### 4.7. Clinical Implications

Our findings indicate that neutrophil recovery after BSI in acute leukemia is shaped by the broader treatment and clinical context in which infection occurs. Recorded G-CSF exposure was consistently associated with a higher cumulative incidence of recovery, but this observational association should not be interpreted as evidence of a causal treatment effect. Treatment context, including the distinction between sequential antileukemic phases and conditioning for HSCT, and infection severity also contributed substantially to the observed recovery pattern.

In contrast, the strong crude association between antimicrobial resistance and impaired recovery was markedly attenuated after adjustment, suggesting that resistance alone provides limited independent information regarding subsequent neutrophil recovery once treatment context and clinical severity are considered. Polymicrobial BSI may identify an additional high-risk phenotype, but this finding requires confirmation.

Competing-risk analysis provides a clinically relevant framework for studying neutrophil recovery after severe infection because death can preclude observation of recovery. Future multicenter studies incorporating detailed leukemia status, treatment exposure, supportive care, and time-dependent clinical variables are needed to determine whether these findings can be reproduced and translated into individualized estimates of hematopoietic recovery after BSI. Such work is particularly relevant as modern AML therapy becomes increasingly heterogeneous and dependent on treatment-specific supportive-care strategies [31].

## 5. Conclusions

In adults with acute leukemia and microbiologically documented BSI, neutrophil recovery was associated more strongly with treatment and clinical context than with antimicrobial resistance itself. Consolidation treatment context and recorded G-CSF exposure were associated with a higher cumulative incidence of recovery in adjusted competing-risk analyses, whereas the crude adverse association with clinically relevant resistance was substantially attenuated after adjustment. The G-CSF finding should be interpreted as an observational association rather than evidence of treatment efficacy because exposure was clinically determined and could not be modeled according to exact timing, dose, duration, or indication. These findings support evaluation of neutrophil recovery in a framework that accounts for competing mortality and recurrent infections and require confirmation in larger multicenter cohorts.

## Figures and Tables

**Figure 1:**
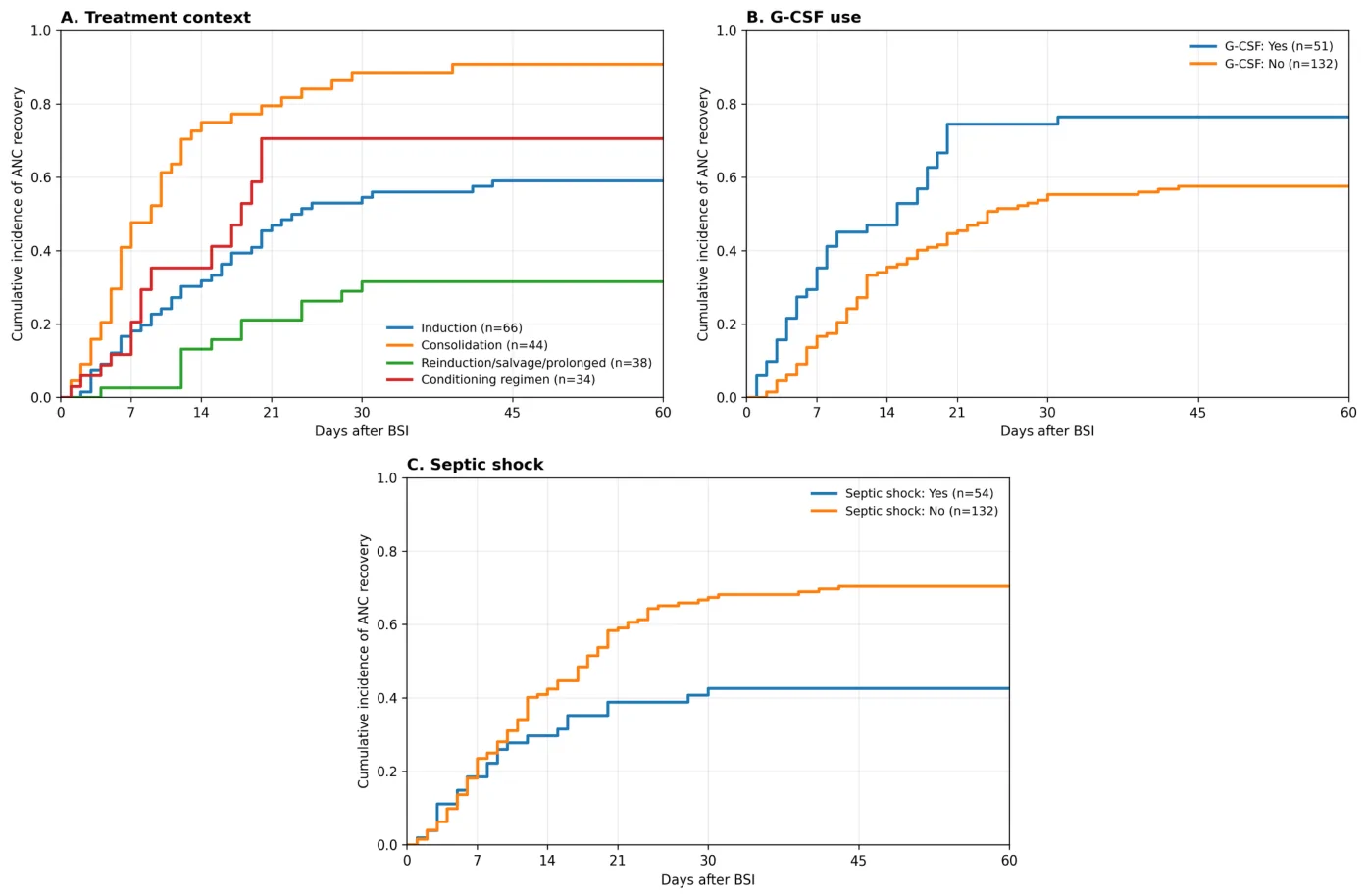
Descriptive cumulative incidence of ANC recovery after bloodstream infection. (**A**) Treatment context: induction, consolidation, reinduction/salvage/prolonged therapy, and conditioning. (**B**) G-CSF use: episodes with versus without G-CSF exposure. (**C**) Septic shock: episodes with versus without septic shock. Panels were enlarged and rearranged to improve readability. Death before ANC recovery was treated as the competing event. Curves are descriptive episode-level cumulative-incidence displays. Formal inference accounting for recurrent BSI episodes was based on the fully adjusted patient-clustered Fine–Gray model (Table 2): consolidation vs induction, *p* = 0.001; reinduction/salvage/prolonged therapy vs induction, *p* = 0.135; conditioning vs induction, *p* = 0.280; G-CSF exposure, *p* = 0.006; and septic shock, *p* = 0.414. Thus, the *p*-values refer to clustered competing-risk regression rather than unadjusted curve-comparison tests. Abbreviations: ANC, absolute neutrophil count; BSI, bloodstream infection; G-CSF, granulocyte colony-stimulating factor.

**Table 1:** Clinical and microbiological characteristics of bloodstream infection episodes according to neutrophil-recovery outcome.

Characteristic	Category	Overall Cohort (*n* = 205)	Endpoint-Evaluable (*n* = 186)	ANC Recovery (*n* = 116)	Death Before Recovery (*n* = 70)
Diagnosis	AML *	168 (82.0)	155 (83.3)	92 (79.3)	63 (90.0)
Diagnosis	ALL	37 (18.0)	31 (16.7)	24 (20.7)	7 (10.0)
Treatment context	Induction	72 (36.4%)	66 (36.3%)	39 (33.9%)	27 (40.3%)
Treatment context	Consolidation	52 (26.3%)	44 (24.2%)	40 (34.8%)	4 (6.0%)
Treatment context	Reinduction	20 (10.1%)	20 (11.0%)	8 (7.0%)	12 (17.9%)
Treatment context	Salvage therapy	11 (5.6%)	11 (6.0%)	3 (2.6%)	8 (11.9%)
Treatment context	Conditioning regimen	34 (17.2%)	34 (18.7%)	24 (20.9%)	10 (14.9%)
Treatment context	Prolonged therapy	9 (4.5%)	7 (3.8%)	1 (0.9%)	6 (9.0%)
Clinical features	Septic shock	57 (27.8%)	54 (29.0%)	23 (19.8%)	31 (44.3%)
Clinical features	MODS	30 (14.6%)	30 (16.1%)	10 (8.6%)	20 (28.6%)
Clinical features	G-CSF use	55 (27.6%)	51 (27.9%)	39 (33.9%)	12 (17.6%)
Clinical features	CRP, mg/dL, median (IQR)	8.4 (4.2–14.9); *n* = 196	8.5 (4.2–16.2); *n* = 182	5.7 (3.1–11.2); *n* = 114	13.4 (8.8–24.1); *n* = 68
Microbiology	Clinically relevant resistant phenotype	54 (26.3%)	51 (27.4%)	22 (19.0%)	29 (41.4%)
Microbiology	Polymicrobial BSI	33 (16.1%)	32 (17.2%)	14 (12.1%)	18 (25.7%)
Microbiology	Gram-negative only	91 (45.5%)	83 (45.1%)	52 (45.2%)	31 (44.9%)
Microbiology	Gram-positive only	84 (42.0%)	77 (41.8%)	54 (47.0%)	23 (33.3%)
Microbiology	Mixed Gram-negative/Gram-positive	20 (10.0%)	19 (10.3%)	6 (5.2%)	13 (18.8%)
Microbiology	Fungal component	5 (2.5%)	5 (2.7%)	3 (2.6%)	2 (2.9%)
Episode characteristics	Recurrent BSI (episode no. ≥ 2)	104 (50.7%)	97 (52.2%)	62 (53.4%)	35 (50.0%)

Values are n (%) unless otherwise indicated. Percentages use available-case denominators. ANC recovery was defined as ANC > 500/µL after BSI; death before recovery was the competing event. Nineteen episodes had an unverifiable recovery endpoint. Acute megakaryoblastic leukemia was classified within AML and was not treated as a separate diagnostic category. Four endpoint-evaluable episodes lacked usable treatment-context coding; three had missing/unclear G-CSF coding; CRP was available in 182/186 endpoint-evaluable episodes. Abbreviations: ALL, acute lymphoblastic leukemia; AML, acute myeloid leukemia; ANC, absolute neutrophil count; BSI, bloodstream infection; CRP, C-reactive protein; G-CSF, granulocyte colony-stimulating factor; HSCT, hematopoietic stem cell transplantation; IQR, interquartile range; MODS, multiple organ dysfunction syndrome. * AML includes the single episode occurring in a patient with acute megakaryoblastic leukemia.

**Table 2:** Multivariable clustered Fine–Gray competing-risk regression for neutrophil recovery after bloodstream infection.

Predictor	M1 sHR (95% CI)	*M1 p*	M2 sHR (95% CI)	*M2 p*	M3 sHR (95% CI)	*M3 p*
Consolidation vs induction	3.21 (1.84–5.62)	<0.001	2.63 (1.53–4.53)	<0.001	2.50 (1.44–4.33)	0.001
Reinduction/salvage/prolonged vs induction	0.47 (0.21–1.05)	0.065	0.55 (0.25–1.22)	0.141	0.54 (0.25–1.21)	0.135
Conditioning vs induction	0.68 (0.26–1.73)	0.415	0.62 (0.26–1.50)	0.291	0.62 (0.26–1.47)	0.280
G-CSF	2.80 (1.24–6.35)	0.014	3.25 (1.42–7.42)	0.005	3.17 (1.40–7.18)	0.006
Septic shock	—	—	0.70 (0.35–1.42)	0.327	0.73 (0.35–1.54)	0.414
CRP per 10 mg/dL	—	—	0.72 (0.53–0.98)	0.036	0.73 (0.53–1.00)	0.053
Clinically relevant resistance	—	—	—	—	0.80 (0.38–1.68)	0.558
Consolidation vs conditioning (additional contrast)	—	—	—	—	4.03 (1.66–9.76)	0.002

M1: treatment context + G-CSF (180 episodes, 94 patients). M2: M1 + septic shock + CRP (177 episodes, 93 patients; 112 recoveries, 65 competing deaths). M3: M2 + clinically relevant antimicrobial resistance (177 episodes, 93 patients; 112 recoveries, 65 competing deaths). sHR > 1 indicates a higher cumulative incidence of recovery in the presence of competing death. Abbreviations: CI, confidence interval; CRP, C-reactive protein; G-CSF, granulocyte colony-stimulating factor; M1–M3, Models 1–3; sHR, subdistribution hazard ratio. The consolidation-versus-conditioning estimate is an additional linear contrast from Model 3 using the same covariates and analytical population; it compares distinct clinical treatment contexts rather than treatment efficacy.

**Table 3:** Sensitivity analyses of the Fine–Gray models for neutrophil recovery after bloodstream infection.

Sensitivity Analysis	Predictor	Episodes, *n*	Patients/Clusters, *n*	sHR	95% CI	*p*
Literal first BSI per patient	Consolidation vs induction	84	84	3.51	1.68–7.35	<0.001
Literal first BSI per patient	Reinduction/salvage/prolonged vs induction	84	84	0.71	0.30–1.65	0.420
Literal first BSI per patient	Conditioning vs induction	84	84	0.60	0.22–1.64	0.321
Literal first BSI per patient	G-CSF use	84	84	4.07	1.83–9.07	<0.001
Literal first BSI per patient	Septic shock	84	84	0.32	0.13–0.78	0.012
Literal first BSI per patient	CRP per 10 mg/dL	84	84	0.88	0.57–1.38	0.580
Literal first BSI per patient	Clinically relevant resistance	84	84	0.52	0.23–1.20	0.125
MODS substituted for septic shock	G-CSF	177	93	3.12	1.42–6.85	0.005
MODS substituted for septic shock	MODS	177	93	0.48	0.14–1.64	0.240
MODS substituted for septic shock	Clinically relevant resistance	177	93	0.73	0.37–1.46	0.376
Polymicrobial BSI substituted for resistance	G-CSF	177	93	3.40	1.56–7.42	0.002
Polymicrobial BSI substituted for resistance	Polymicrobial BSI	177	93	0.50	0.27–0.93	0.028
log2(CRP) instead of linear CRP	G-CSF	177	93	3.16	1.42–7.05	0.005
log2(CRP) instead of linear CRP	log2(CRP)	177	93	0.93	0.85–1.02	0.140
log2(CRP) instead of linear CRP	Clinically relevant resistance	177	93	0.77	0.35–1.68	0.506

Literal-first-BSI analysis: earliest chronological BSI for each of 101 patients; 89 had an evaluable endpoint and 84 had complete M3 covariates (52 recoveries, 32 competing deaths). This analysis used an independent Fine–Gray model. Other sensitivity analyses retained clustered recurrent episodes. For the MODS, polymicrobial-BSI, and log2(CRP) sensitivity models, *n* = 177 episodes from 93 patients (112 recoveries, 65 competing deaths). For compact presentation, only G-CSF and the focal replacement/exposure variables are shown for these three models; all models remained adjusted for treatment context and the other covariates specified in the corresponding primary model. Abbreviations: BSI, bloodstream infection; CI, confidence interval; CRP, C-reactive protein; G-CSF, granulocyte colony-stimulating factor; MODS, multiple organ dysfunction syndrome; sHR, subdistribution hazard ratio.

## Data Availability

The data supporting the findings of this study are available from the corresponding author upon reasonable request, subject to institutional and ethical restrictions related to patient confidentiality.

## Supplementary Materials

The following supporting information can be downloaded at https://www.mdpi.com/article/10.3390/cancers18193234/s1, Table S1. Descriptive comparison of BSI episodes with and without recorded G-CSF exposure. Table S2. Descriptive comparison of endpoint-evaluable and endpoint-unavailable BSI episodes.
