# Supplementary material for: Clinical and Treatment-Related Factors Associated with Neutrophil Recovery After Bloodstream Infection in Acute Leukemia: A Competing-Risk Analysis
Source: Cancers (Basel). 2026 Oct 8;18(19):3234. doi: 10.3390/cancers18193234 (PMC13664072; doi:10.3390/cancers18193234)
Supplement: Supplementary file 1 [file cancers-18-03234-s001.zip › cancers-4601204-supplementary.pdf]

Supplementary Materials

Clinical and Treatment-Related Factors Associated With Neutrophil Recovery After Bloodstream Infection in Acute Leukemia: A Competing-Risk Analysis

Krzysztof Gawronski et al.

Table S1. Descriptive comparison of BSI episodes with and without recorded G-CSF exposure.

Characteristic	G-CSF exposed (n=55)	No G-CSF (n=144)
Treatment context		
Induction	14 (25.5%)	58 (40.3%)
Consolidation	6 (10.9%)	45 (31.3%)
Reinduction/salvage/prolonged	4 (7.3%)	36 (25.0%)
Conditioning for HSCT	31 (56.4%)	2 (1.4%)
Missing treatment context	0	3 (2.1%)
Septic shock	16 (29.1%)	40 (27.8%)
MODS	10 (18.2%)	19 (13.2%)
CRP, median, mg/dL	8.0	8.6
Clinically relevant resistance	13 (23.6%)	39 (27.1%)
Polymicrobial BSI	12 (21.8%)	21 (14.6%)

Values are n (%) unless otherwise indicated. This table is descriptive; no unclustered hypothesis tests were used because recurrent BSI episodes from the same patient are not statistically independent. G-CSF exposure was strongly concentrated in the conditioning/HSCT context.

Table S2. Descriptive comparison of endpoint-evaluable and endpoint-unavailable BSI episodes.

Characteristic	Endpoint evaluable (n=186)	Endpoint unavailable (n=19)
Diagnosis		
AML*	155 (83.3%)	13 (68.4%)
ALL	31 (16.7%)	6 (31.6%)
Treatment context		
Induction	66 (35.5%)	6 (31.6%)
Consolidation	44 (23.7%)	8 (42.1%)
Reinduction/salvage/prolonged	38 (20.4%)	2 (10.5%)
Conditioning for HSCT	34 (18.3%)	0
Missing treatment context	4 (2.2%)	3 (15.8%)
Septic shock	54 (29.0%)	3 (15.8%)
MODS	30 (16.1%)	0
CRP, median, mg/dL	8.5	6.8
Clinically relevant resistance	51 (27.4%)	3 (15.8%)
Polymicrobial BSI	32 (17.2%)	1 (5.3%)

*AML includes acute megakaryoblastic leukemia. Values are n (%) unless otherwise indicated. The comparison is descriptive because the endpoint-unavailable group was small and outcome unavailability cannot be assumed to be random.
